# Transcriptome analysis of genes involved in the pathogenesis mechanism of potato virus Y in potato cultivar YouJin

**DOI:** 10.3389/fmicb.2024.1353814

**Published:** 2024-03-06

**Authors:** Tianqi Yang, Xingyue Zhao, Jinjiang Bai, Wenxia Lv, Qi Chen, Jun Hu, Guangjing Liu, Yuanzheng Zhao, Hongyou Zhou, Mingmin Zhao, Hongli Zheng

**Affiliations:** ^1^College of Horticulture and Plant Protection, Inner Mongolia Agricultural University, Hohhot, China; ^2^Inner Mongolia Zhongjia Agricultural Biotechnology Co., Siziwang Banner, China; ^3^Siziwang Banner Agricultural and Livestock Products Quality and Safety Inspection and Testing Station, Siziwang Banner, China; ^4^Inner Mongolia Academy of Agricultural and Animal Husbandry Sciences, Hohhot, China; ^5^Key Laboratory of the Development and Resource Utilization of Biological Pesticide in Inner Mongolia, Hohhot, China

**Keywords:** potato virus Y, potato cultivar YouJin, RNA-Seq, functional analysis, candidate genes

## Abstract

**Introduction:**

Potatoes (*Solanum tuberosum* L.) can be infected by various viruses, but out of all of viruses, the potato virus Y (PVY) is the most detrimental. Research shows that the potato cultivar YouJin is especially vulnerable to PVY and displays severe symptoms, including leaf vein chlorosis, curled leaf margins, large necrotic spots on the leaf blades, and the growth of small new leaves.

**Methods:**

PVY infection in potato cultivar YouJin was confirmed through symptom observation, RT-PCR, and Western blot analysis. Transcriptome sequencing was used to analyze the genes associated with PVY pathogenesis in this cultivar.

**Result:**

Transcriptome analysis of differential genes was conducted in this study to examine the pathogenesis of PVY on YouJin. The results showed that 1,949 genes were differentially regulated, including 853 upregulated genes and 1,096 downregulated genes. The Gene Ontology (GO) and Kyoto Encyclopedia of Genes and Genomes (KEGG) enrichment analysis indicated that carbohydrate synthesis and metabolism pathways were suppressed, and electron transferase and hydrolase activities were reduced. Moreover, there were increased expression levels of protein kinase genes. By focusing on plant–pathogen interaction pathways, six core genes all upregulating the WARK family of transcription factors were obtained. Additionally, a constructed PPI network revealed the identification of key modular differential genes, such as downregulated photosynthesis-related protein genes and upregulated AP2/ERF-ERF transcription factors. Functional network enrichment analysis revealed that PVY infection limited RNA metabolism, glutathionylation, and peroxiredoxin activity while triggering the expression of associated defense genes in YouJin. After analyzing the above, 26 DEGs were screened and 12 DEGs were confirmed via RT-qPCR.

**Conclusion:**

These results establish a hypothetical framework for clarifying the pathogenesis of PVY in the YouJin variety of potatoes, which will help design the disease resistance of YouJin.

## 1 Introduction

In recent years, potato (*Solanum tuberosum* L.) has played an important role as a food crop in ensuring food security at the national level (Sajeevan et al., 2023). Increased consumption of potato in Asian countries such as China and India is the main driver of its overall consumption growth (Shahbandeh, 2023). However, plant growth can be influenced by various biotic and abiotic stresses, such as pests, weeds, viruses, drought, and salinity, which restrict crop growth and yield. Among the various factors that impact the growth of potatoes, viral diseases are the most prevalent (Wang et al., 2011; Lal et al., 2017). Of the various plant viruses, potato virus Y (PVY) is a member of the *Potyvirus* genus, which belongs to the family *Potyviridae*. It is also one of the viruses responsible for significant economic losses in the cultivation of potatoes (Lacomme et al., 2017).

PVY can be transmitted by more than 50 species of aphids, as well as by machinery such as large agricultural equipment and tools. PVY in potatoes usually causes symptoms of stem vein necrosis, mottling, yellowing of leaflets, defoliation, and plant dwarfing (Slater et al., 2020). Viral infections not only impact the host’s normal physiological processes but also utilize host proteins to replicate and proliferate, resulting in changes to host gene expression profiles (Sambade et al., 2003). Transcriptome sequencing can be used to analyze both gene expression and transcription factors in a comprehensive manner, which uncovers genes that affect particular biological processes (BPs) and disease development (Vives et al., 2005; Satoh et al., 2010). For instance, transcriptome sequencing analysis of PVY-sensitive and PVY-resistant varieties revealed enrichment of differential genes in photosynthesis within the GO functional classification. The primary differential genes encode ABC transporter proteins and non-specific lipid transfer proteins (nsLTPs). It is worth noting that nsLTPs belong to the PR-14 family of proteins, which have been linked to the disease’s pathogenesis (Goyer et al., 2015). The variable splicing analysis of PVY-resistant and PVY-susceptible varieties revealed that genes encoding proteins of the polynucleotidyl transferase and ribonuclease H-like family, also known as Werner syndrome-like exonucleases (WEXs), had the most statistically intensive variable splicing transcripts. Certain WEX proteins play a critical role in the function of the RNA-induced silencing complex (RISC) (Ross et al., 2022). The results from transcriptomic studies of the thermoregulatory mechanisms governing plant–virus interactions suggest that the over-accumulation of ADP-ribose [poly(adenosine diphosphate-ribose), PAR] caused by PVY infection may play an essential role in the response of potatoes to PVY (Glushkevich et al., 2022).

This study used the transcriptome sequencing technology to analyze the differential gene expression in the disease-susceptible variety YouJin after PVY infestation. To screen the important genes, the annotation analysis of significant differential expression of genes, the focus on key KEGG pathways, and the construction of the PPI interactions network of all the differential genes were analyzed. This study provides a preliminary exploration of the pathogenesis of PVY on YouJin, laying the foundation for subsequent improvement of the potato cultivar YouJin.

## 2 Materials and methods

### 2.1 Experimental materials

The potato varieties YouJin and PVYros were provided by Inner Mongolia Zhongjia Biotechnology Co. and Jose Antonio Daros, Spain, respectively.

### 2.2 Potato plant preparation

Potato plants were cultured on an MS medium containing 3% sucrose in a light incubator at 22°C under a 16/8-h light/dark cycle. Potato seedlings, grown for 3 weeks, were transferred to plastic pots (10 × 10 cm) filled with nutrient soil (comprising a 3:1 ratio of soil and vermiculite). They were then placed in an artificial climate chamber for 16 h of light incubation at 22°C and 8 h of dark incubation at 16°C until they had grown six true leaves, at which point they were deemed ready for use.

### 2.3 Inoculation of PVYros

PVYros was inoculated into *Nicotiana benthamiana* (*N. benthamiana*) plants, and, after disease onset, leaves from plants showing obvious symptoms were collected for virus identification. Leaves from susceptible plants were stored in the laboratory at −80°C in a refrigerator. A volume of 4 mL of phosphate buffer (PB, pH = 7.2) was added to 2 g of leaf tissue removed from the refrigerator, crushed quickly, vortexed on an oscillator to mix well, and placed on ice. The viral crude extract of leaves (20 μL) was inoculated on *N. benthamiana* leaves sprayed with 600 mesh quartz sand using the traditional sap rubbing inoculation method. A control group in good health was established, with secondary inoculation taking place 3 days later. Diseases and symptoms were then observed 14 days later, and systematic leaves were collected from both susceptible and healthy control plants.

The plant leaves were snap-frozen in liquid nitrogen after inoculation at 30 dpi for total RNA extraction and detection. The upper leaves were collected for transcriptome sequencing. Three biological replicates were established for PVY-infected plants (YJ-PVY1, YJ-PVY2, and YJ-PVY3) and healthy plants (YJ-H1, YJ-H2, and YJ-H3).

### 2.4 RT-PCR and Western blot for virus detection

The cDNA reverse transcription kit, Evo M-MLV Plus cDNA, was obtained from Accurate Biotechnology Co., Ltd., Hunan, China. The cDNA was generated using Evo M-MLV Plus RTase reverse transcriptase by Random Primers. Based on the gene sequence of PVY coat protein (CP) registered in GenBank (GenBank: MN563134.1), the corresponding primers were designed as PVY CP-F (5′–3′): GCAAATGACACAATTGATGC and PVY CP-R (5′–3′): CATGTTCTTGACTCCAAGTAG. CP-F and PVY CP-R were used as primers for PCR. PCR amplification was performed according to the rTaq instructions of Takara Biomedical Technology Co., Ltd. The plant samples are ground in a pre-cooled mortar and pestle and collected in 1.5 mL centrifuge tubes. Protein lysate was added to the centrifuge tube in a 2:1 ratio, and the mixture was incubated in a 95°C water bath for 10 min, followed by 2 min on ice. The centrifuge tubes were then spun at 12,000 rpm for 10 min at 4°C. A total of 10–20 μL of the prepared protein samples were used for SDS-PAGE electrophoresis. Following the completion of electrophoresis, the membrane transfer process was initiated. Subsequently, the membrane was incubated with a 1,000-fold diluted PVY CP monoclonal antibody and a 5,000-fold diluted alkaline phosphatase-labeled sheep anti-rabbit IgG secondary antibody in the blocking solution. The nitrocellulose membrane was washed with 1 × TBST and then treated with an ECL color development solution (Bio-Rad, United States) to visualize the protein bands on a fluorescence and chemiluminescence dual-function imaging system.

### 2.5 Total RNA extraction and transcriptome sequencing

Total RNA was extracted using the TRIzol method (please refer to the instruction manual of the TRIzol kit from Beijing Tiangen Deepening Technology Co., Ltd. for specific details). The transcriptome analysis of YouJin following PVY infestation was performed by Beijing Biomaker Biotechnology Co. The transcriptome comprised six cDNA libraries, consisting of three biological replicates and two sets of treatments. Following successful quality control, the libraries were sequenced using the Illumina NovaSeq6000 in PE150 mode. Trimmomatic (Bolger et al., 2014) was used to remove the adapter from FASTQ sequences on the Illumina platform, which trims the FASTQ sequence file based on base quality values. Data quality was ensured by filtering out ligated and low-quality reads (including the removal of reads with greater than 10% N and more than 50% of the entire read in bases with a quality value of Q ≤ 10), resulting in high-quality clean sequences. The reference genome version used in this project was *Solanum tuberosum*.v4.03.genome.fa. The clean reads were compared to the reference genome quickly and accurately using HISAT2 (Kim et al., 2015) software to obtain the localization information of the reads on the reference genome. StringTie (Pertea et al., 2015) was used to assemble the reads on comparison for transcriptome reconstruction, facilitating subsequent analysis.

### 2.6 Annotation of differential gene expression and analysis

Transcript and gene expression levels were measured using FPKM. DESeq2 (Love et al., 2014) software was utilized for differential expression analysis between sample groups with biological replicates. During differential gene expression analysis, the fold change (FC) denotes the expression ratio between two groups. The false discovery rate (FDR) is a corrected value that confirms the significance of the difference by using the value of p. The criteria for screening genes for their significance were set using FC ≥ 2 and FDR < 0.01. A sequence comparison of differentially expressed gene (DEG) sequences with NR, Swiss-Prot, COG, KOG, and KEGG databases was carried out through DIAMOND (Buchfink et al., 2015) software, thereby attaining the relevant annotation information for KEGG differential genes. The obtained GO function enrichment information of differential genes was analyzed through InterProScan (Jones et al., 2014) software, using the database integrated by InterPro to assess the GO orthology results. The regulatory genes responsible for differential gene expression were predicted using the PlantTFDB (Jin et al., 2017) database to identify transcription factor families. The heat map was drawn using ChiPlot^1^ (accessed on 10 August 2023). The study analyzed the protein interactions of the differential genes using the STRING database (Szklarczyk et al., 2023) and Cytoscape (Shannon et al., 2003), constructed PPI networks for all 1,949 differential genes and obtained the most important modules using MCODE, and further analyzed the functional networks of the differentially upregulated and downregulated genes using ClueGO in Cytoscape.

### 2.7 RT-qPCR validation of differentially expressed genes

Twelve DEGs of YouJin were selected to undergo RT-qPCR validation following infestation by PVY. The internal reference gene was NtUB (N-Tub, ID:394889), and the necessary primers were synthesized by Shanghai Sangon Biological Co. (Supplementary Table S1). The cDNA templates were obtained from transcriptome sequencing samples. Each treatment had three biological and four technical replicates. The three biological replicates of the experimental group were YJ-PVY1, YJ-PVY2, and YJ-PVY3, and the three biological replicates of the control group were YJ-H1, YJ-H2, and YJ-H3. Experimental and control groups with three biological replicates each were considered as one set of technical replicates. The 2^−ΔΔCT^ method was used to determine the relative expression.

## 3 Results

### 3.1 Symptoms of PVYros infection in potato cultivar YouJin and virus detection

**Figure 1:**
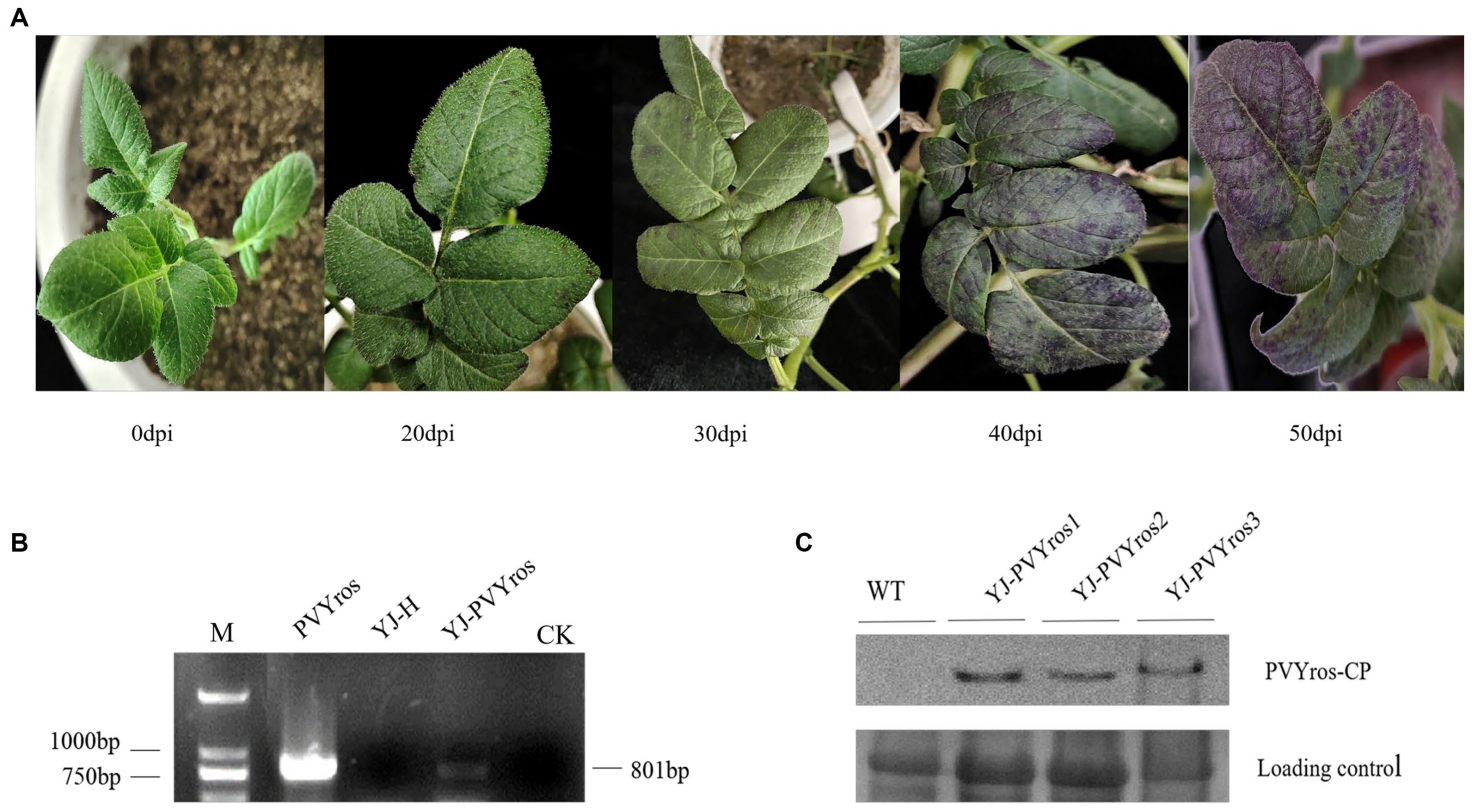
Symptoms of PVYros infection in YouJin. **(A)** Symptoms of PVYros infestation; **(B)** RT-PCR virus detection; and **(C)** viral accumulation were detected by Western blotting.

Potato plants grown to six true leaves were inoculated with PVYros via rubbing. As shown in Figure 1A, observations were made for symptoms on potato plants 20 days post-inoculation. In comparison to the healthy control, the leaves of PVYros-inoculated plants exhibited distinct purple spots. Between 30 and 40 days post-inoculation, PVYros leads to the spread of purple blemishes across the whole leaf, causing severe symptoms such as crumpling, distortion, and margin down turning. In advanced PVY infestations, new leaf growth on plants exhibited purple spots and stunted growth. PVY positivity was detected by RT-PCR in the leaves of plants with significant (Figure 1C). The PVY-specific antibody was utilized to identify viral accumulation in samples at 30 dpi through Western blotting analysis. The results demonstrate that the CP of PVYros was not detected in the healthy control group, but it was found in all three sets of biological replicas from samples that were inoculated with PVYros in potato cultivar YouJin leaves (Figure 1B). Thus, this validates a successful PVY infection in potatoes.

### 3.2 Transcriptome sequencing of potato cultivar YouJin infected with PVYros

We conducted transcriptome sequencing on six samples of the potato cultivar YouJin infected with PVY. We obtained a total of 39.72 GB of clean data. The G + C content of the bases was over 42%, and the proportion of Q30 bases was over 95.93% (Table 1). These findings confirm the accuracy and reliability of the results obtained through sequencing assembly, making them suitable for further analysis. As shown in Table 1, the comparison efficiency of all samples’ reads with the reference genome was observed to range from 85.04 to 88.61%. The three PVY-infected libraries showed a comparison rate between 85 and 88%, while the comparison rate of control libraries was slightly higher, ranging from 87 to 88%. Furthermore, the multi-locus matching rate of the samples was less than 0.12%, affirming that the reference data provided a credible basis for further analysis.

**Table 1:** RNA-Seq data and sequence comparison results of sample sequencing data with the reference genome.

Samples name	YJ-PVY1	YJ-PVY2	YJ-PVY3	YJ-H1	YJ-H2	YJ-H3
Q30	96.92%	96.97%	96.33%	96.36%	96.97%	95.93%
GC content	43.59%	43.05%	42.61%	43.06%	42.39%	42.88%
Clean bases	6.51E+09	6.56E+09	6.14E+09	6.35E+09	7.05E+09	7.12E+09
Total clean reads	2,17,77,713	2,19,46,690	2,05,23,235	2,12,83,720	2,35,67,153	2,38,07,726
Total reads	4,25,67,440	4,25,67,440	4,25,67,440	4,38,93,380	4,25,67,440	4,25,67,440
Mapped reads	3,85,93,254	3,49,05,682	4,01,89,494	3,87,80,971	3,76,56,546	4,18,32,864
Mapping rate	88.61%	85.04%	85.27%	88.35%	88.46%	87.86%
Uniq mapped reads	3,47,50,097	3,37,03,274	3,87,34,056	3,72,68,776	3,61,96,571	4,01,59,095
Multiple map reads	38,43,157	12,02,408	14,55,438	15,12,195	14,59,975	16,73,769
Multiple map rate	8.82%	2.93%	3.09%	3.45%	3.43%	3.52%

**Figure 2:**
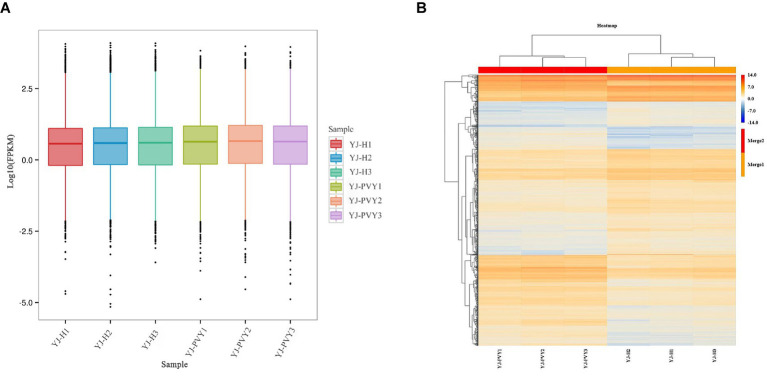
Gene density of the cDNA library and clustering heat map of gene expression. **(A)** Gene density of cDNA libraries and **(B)** a clustering heat map of gene expression. Yellow and blue colors indicate upregulation and downregulation of genes.

The median line of the experimental group was slightly higher than that of the healthy control group, indicating an increase in gene expression after inoculation with PVYros. Meanwhile, the sizes of the six boxes were similar, suggesting a high similarity in gene density between the six samples. The relative consistency in the length of the whisker lines is an indication of their similar variances. The median line of the samples is closer to the high value of the box, and the long whiskers extend toward the low value, resulting in a negative bias (Figure 2A). According to hierarchical cluster analysis, both PVY-infected and healthy samples exhibited a good correlation. As displayed in the heat map, the FC of the gene exhibited greater consistency among the three biological replicates of PVY inoculation and the three replicates of the healthy control group (Figure 2B), thus affirming the reliability of the data and enabling its use for further analyses.

### 3.3 Differential gene expression analysis and screening for significantly expressed differential genes of the potato cultivar YouJin infected with PVYros

**Figure 3:**
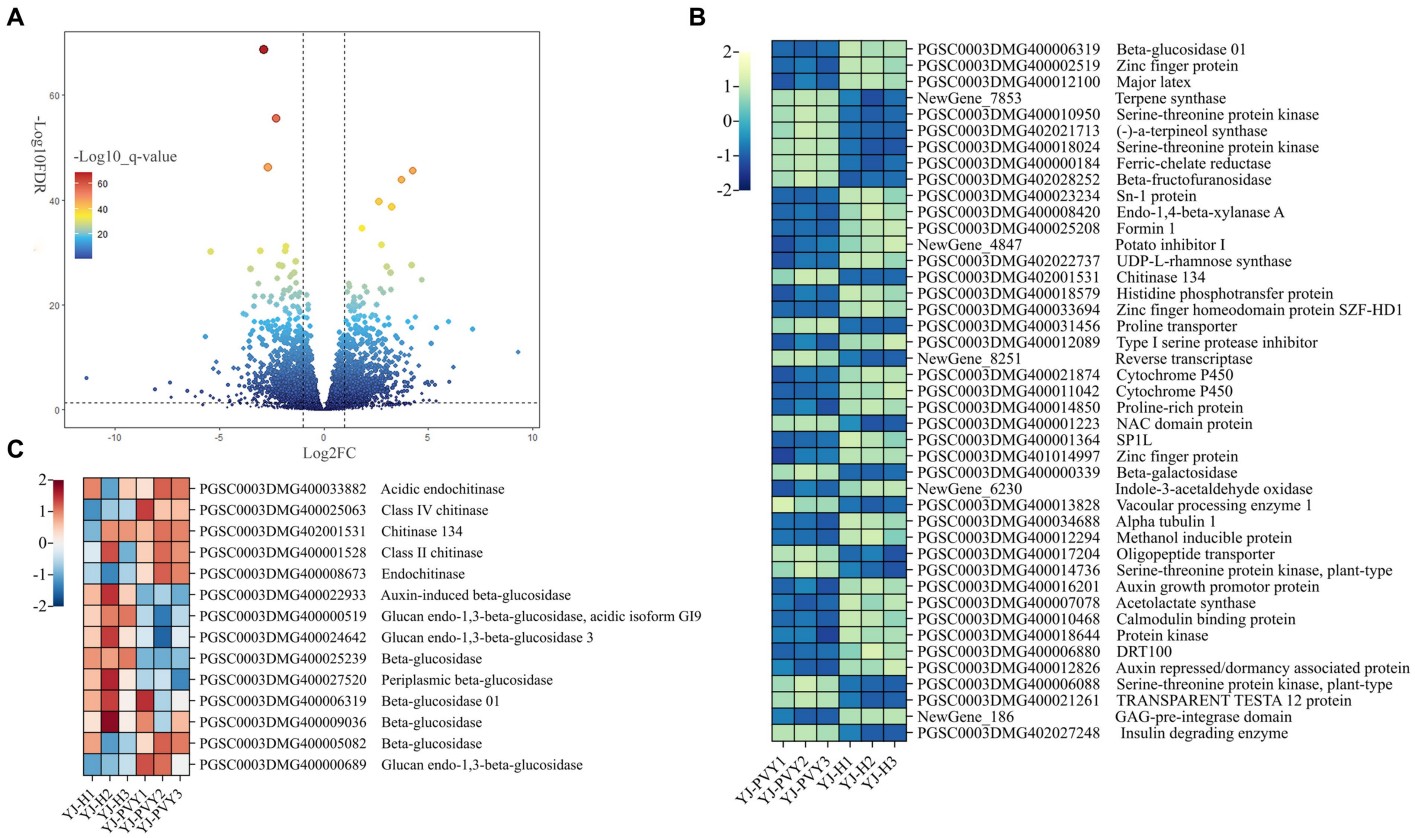
Volcano plots and a clustering heat map of the differentially expressed genes. **(A)** A volcano map of all genes; **(B)** a heat map for genes of significantly differential expression; yellow and blue colors indicate genes of upregulation and downregulation; and **(C)** a heat map for gene clusters of chitinase and glucosidase; red and blue colors indicate genes of upregulation and downregulation.

A total of 1,949 genes exhibiting differential expression were identified following PVYros infection (Figure 3A; Supplementary Table S2), with 853 upregulated and 1,096 downregulated genes, using healthy potatoes as the control group. As shown in Figure 3B, we identified genes related to PVYros response that were significantly differentially expressed (Log_2_FC > ±2, −Log_10_FDR > 21) through preliminary screening (Supplementary Table S3). Using gene annotations from NR and Swiss-Prot database analysis, we found that PVYros infection affects the regulation of sugar metabolism responsible for plant growth and development. Specifically, the gene expression of glucosidase was significantly suppressed (Figure 3B). However, many studies have shown that the synthesis of glucan endo-1,3-beta-glucosidase is stimulated when plants are infected by viral pathogens, and its concentration also increases dramatically. Glucan endo-1,3-beta-glucosidase appears to be coordinately expressed along with chitinases after fungal infection (Kebede and Kebede, 2021). Then, we screened a total of nine glucosidase genes among all DEGs, of which seven were downregulated and two were upregulated (Figure 3C). Simultaneously, to resist PVY infection, the expression of certain potato disease-resistance genes was strongly promoted, including significant increases in chitinases (Zhao et al., 2023) and NAC structural domain proteins (Chang et al., 2020). At the same time, we observed upregulation of all five chitinase genes among the overall DEGs (Figure 3C). This finding indicates that the expression of important genes for plant growth and development was suppressed, and genes related to plant disease resistance were activated upon PVY infestation.

Upon conducting GO analysis (Table 2) on the initially screened DEGs (Figure 3B), terms chosen for gene annotation include metabolic process (GO:0008152), biological regulation (GO:0065007), response to stimulus (GO:0050896) in BP and transporter activity (GO:0022857), binding (GO:0005488), and molecular function (MF) regulator (GO:0098772), resulting in a total of 25 differential genes (Supplementary Table S4). After removing functionally redundant genes, the major genes included serine–threonine protein kinase (STE), major latex protein (MLP), TRANSPARENT TESTA 12 protein (TT12), beta-fructofuranosidase (β-FFase), Chitinase 134 (Chi134), auxin growth promoter protein (AuP), cytochrome P450 (CYP450), beta-glucosidase (GUS), alpha-tubulin 1 (TUBA1A), histidine phosphotransfer protein (Hpt), type I serine protease inhibitor (PIs1), oligopeptide transporter (OPT), acetolactate synthase (AHAS), protease inhibitor (PI), and GAG-pre-integrase domain (GAG), proline transporter (PRT), calmodulin-binding protein (CBP), GDSL-like lipase (GDSL), and DNA damage repair/toleration protein (DRT).

**Table 2:** GO, KEGG and transcription factors analysis of major differential genes.

Category	Term	Counts
BP	Metabolic process	16
	Cellular process	12
	Localization	1
	Biological regulation	3
	Response to stimulus	4
CC	Cellular anatomical entity	14
	Intracellular	3
MF	Catalytic activity	21
	Binding	15
	Transporter activity	1
	Molecular function regulator	1
KEGG pathway	Plant-pathogen interaction	4
	MAPK signaling pathway—plant	4
	Plant hormone signal transduction	2
	Amino sugar and nucleotide sugar metabolism	2
	Galactose metabolism	2
	Monoterpenoid biosynthesis	2
Transcription factor	RLK-Pelle_LRR-XII-1	1
	zf-HD	1
	NAC	1
	TKL-Pl-4	1
	RLK-Pelle_LysM	1
	AGC_PKA-PKG	1

The genes that were differentially expressed during the initial screening underwent a KEGG pathway analysis (Table 2). From this analysis, differential genes that were associated with the plant–pathogen interaction signaling pathway (ko04626), MAPK signaling pathway (ko04016), and plant hormone signal transduction pathway (ko04075) were selected. A total of seven genes (Supplementary Table S5) were obtained, and functional redundancy genes were excluded. Ultimately, three differential genes were found to be STE, Chi134, and Hpt, which overlapped with the key GO genes. It was hypothesized that the aforementioned DEGs that overlap may have a significant impact on the development of PVY infestations in potatoes.

Predictions of transcription factor families that exhibit significant expression of differential genes were made using the PlantTFDB database (Supplementary Table S6). As shown in Table 2, the genes with differential expression in the primary screening underwent further analysis to predict their transcription factor families. Consequently, six genes were annotated, and one differential gene was identified as the NAC domain protein gene, regulated by the NAC transcription factor family. It is posited that this gene activates NAC transcription factors to defend against the virus following PVY infestation in YouJin, impacting plant growth, development, and morphological changes (Shao et al., 2015).

### 3.4 GO and KEGG enrichment analyses of differentially expressed genes in potato cultivar YouJin infected with PVYros

**Figure 4:**
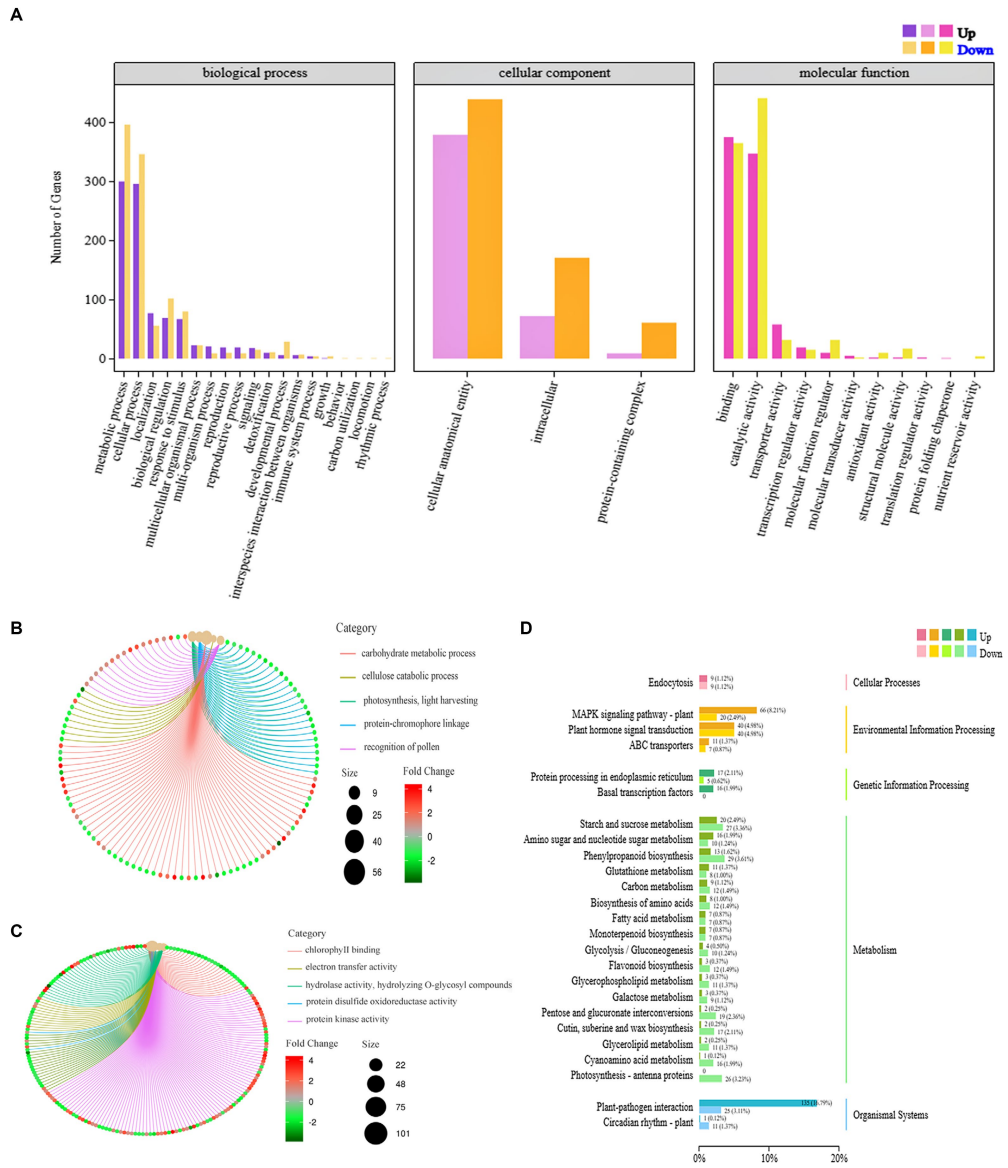
Differential gene GO functional enrichment analysis. **(A)** GO functional annotation classification statistical map of differential genes; **(B)** a network diagram of biological process; **(C)** a network diagram of molecular function enrichment; and **(D)** a KEGG pathway classification map.

The DEGs derived from the analysis underwent GO function enrichment analysis. The statistics of the GO annotation classification are presented in Figure 4A, revealing a significant enrichment of 33 gene functions (with a *Q* ≤ 0.05). In the BP branch, the two subclasses containing the most DEGs were metabolic processes and cellular processes; in the cellular component (CC) branch, the three subclasses containing DEGs were cellular anatomical entities, intracellular parts, and protein-containing complex parts. In the MF branch, the subclasses containing the most DEGs were binding, catalytic activity, and transporter activity. In BPs, the GO terms enrich the bioregulation, detoxification, and immune system processes, implying that PVY infestation triggered the active expression of cellular defense-related genes in YouJin. The network maps were analyzed for key functional enrichments of BPs and molecular functional branches of the most enriched differential genes. As shown in Figure 4B, the majority of genes exhibited enrichment in the carbohydrate pathway with downregulated expression, whereas all differential genes related to photosynthesis were also downregulated. Additionally, Figure 4C shows a significant suppression of chlorophyll-binding protein function, along with the decreased activity of electron transferase and hydrolase. This finding was contrasted by an overall increase in protein kinase gene expression levels.

The findings of KEGG signaling pathway enrichment are shown in Figure 4D. It was discovered that the genes with the highest level of enrichment were associated with plant–pathogen interaction and the plant MAPK signaling pathway. Moreover, there was a higher number of upregulated genes compared to downregulated genes in two pathways, potentially involved in virus resistance. In terms of metabolism-related pathways, there were more downregulated genes than upregulated genes, suggesting that significant metabolic pathways related to life activities were inhibited after PVY infestation in potato cultivar YouJin.

**Figure 5:**
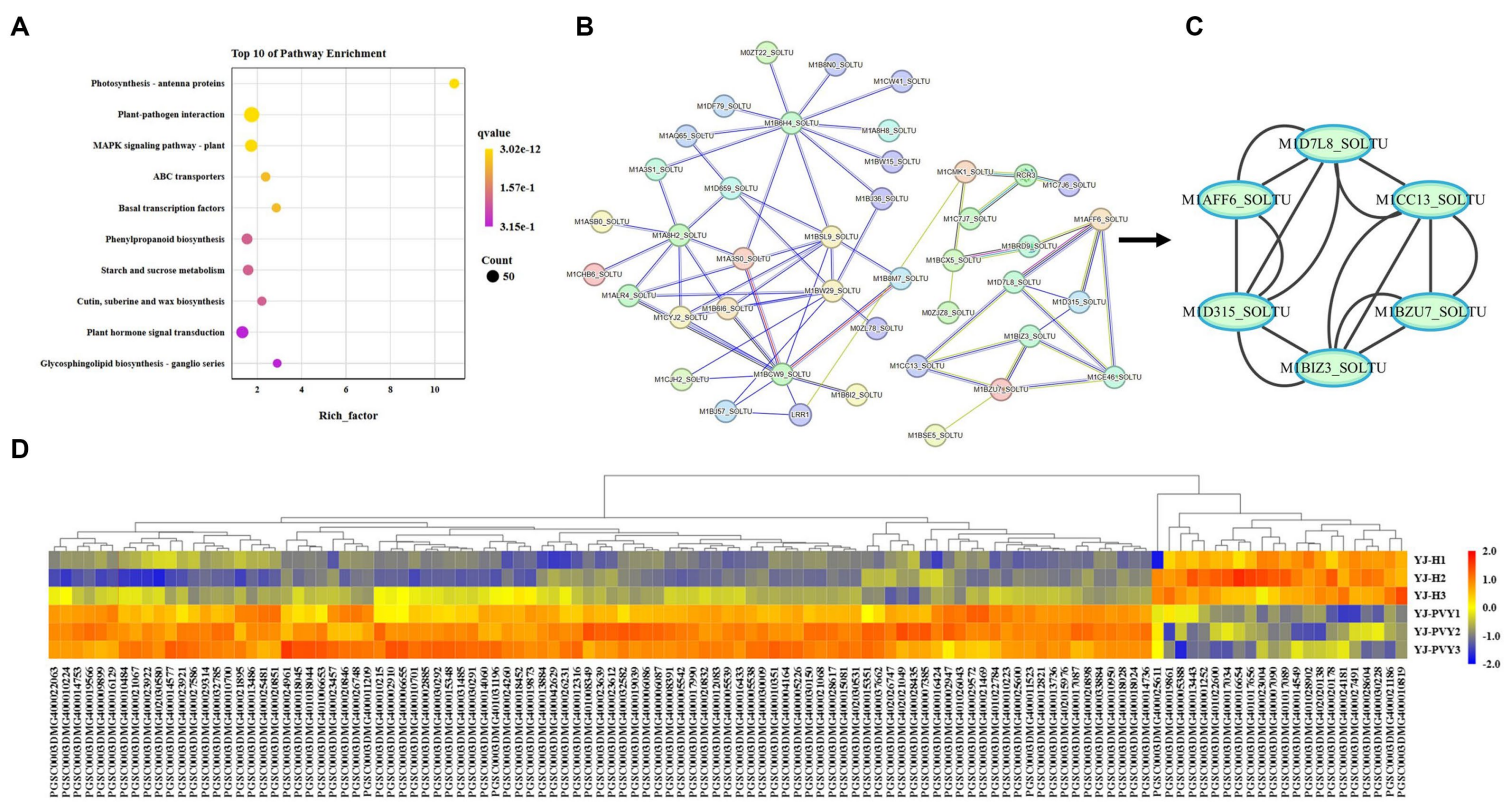
KEGG pathway enrichment analysis of the integrated DEGs. **(A)** The top 10 KEGG pathway enrichments. **(B)** PPI network analysis of plant–pathogen interactions based on the string database. **(C)** Key module genes in the network are ranked by the degree method using MCODE. **(D)** A heat map of the DEGs enriched in the PPI interactions. The expression values of the six libraries are shown as normalized FPKM values. Red colors indicate upregulated DEGs. Blue colors indicate downregulated DEGs.

Among the top 10 pathways with the highest enrichment, the plant–pathogen interaction pathway had the highest enrichment (Figure 5A). We focused on plant–pathogen interaction pathways that are not isolated but interconnected through a complex regulatory network. These pathways involve a large number of regulatory factors and genes, which constitute a complex regulatory network (Ding et al., 2022). Thus, we analyzed the differential genes involved in plant–pathogen interactions through protein interaction analysis (Figure 5B). The PPI enrichment *p*-value was found to be less than 1.0e-16. The PPI network comprised a total of 120 DEGs (Supplementary Table S8) in the plant–pathogen interactions pathway, of which 99 were upregulated genes and 21 were downregulated genes (Figure 5D). The key module genes in WRKY transcription factor were identified using MCODE in Cytoscape (Figure 5C), including M1BIZ3_SOLTU(PGSC0003DMG400017990), M1CC13_SOLTU(PGSC0003DMG400024961),

M1D315_SOLTU(PGSC0003DMG401031196), M1AFF6_SOLTU(PGSC0003DMG400008391), M1BZU7_SOLTU(PGSC0003DMG400022063), and M1D7L8_SOLTU(PGSC0003DMG400033884).

### 3.5 PPI network construction and genetic analysis of key modules and functional network analysis of DEGs in potato cultivar YouJin infected with PVYros

**Figure 6:**
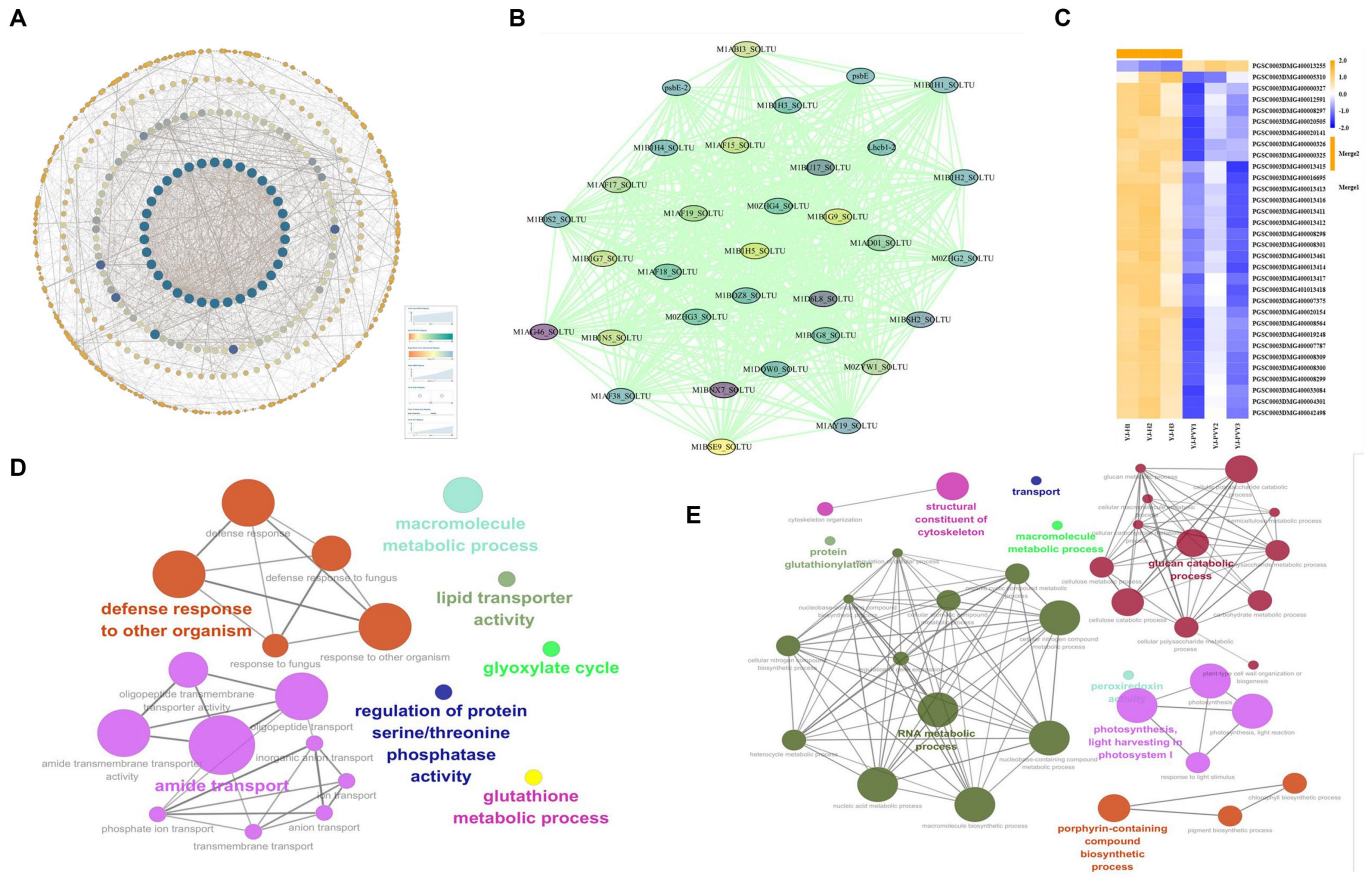
Analysis of PPI networks and functional network analysis of differentially expressed genes (DEGs) reveals the grouping of terms, with nodes connected according to their kappa score (≥0.3). **(A)** PPI network; **(B)** key modular genes by MCODE; **(C)** a heat map of the key module genes; blue indicates lower expression, and yellow indicates higher expression. **(D)** Functional network analysis of upregulated DEGs; **(E)** functional network analysis of downregulated DEGs.

The PPI network of all differential genes was created through an analysis of 1,949 differential gene–protein interactions using the STRING database and Cytoscape (Figure 6A). The most significant modules were then obtained by using the MCODE application in Cytoscape (Figure 6B). The results indicated that the PPI network consisted of 33 nodes and 519 edges, which demonstrated a significantly higher number of interactions than expected, with a PPI-enriched value of p of <1.0E-16. Functional annotations of key protein genes are listed in Supplementary Table S7 and Figure 6C, showing that 31 downregulated genes were mainly light-harvesting complex (LHC) proteins, and the blocked pathway of plant light-harvesting may be the main factor leading to the new leaf size in the late stage of the disease, which suggests that the PVY infestation severely hinders photosynthesis in YouJin. A gene was also found upregulated for AP2/ERF-ERF transcription factor, and the mechanism of photosynthesis in its relevance is not clear, but some studies suggest that AP2/ERF-ERF transcription factors play a role in resisting pathogen invasion (Chacón-Cerdas et al., 2020).

Functional network analyses of differentially upregulated and downregulated genes were conducted separately through the use of ClueGO in Cytoscape. As shown in Figure 6D, the analysis of the upregulated genes showed the presence of seven functional clusters, including defense response to other organisms, amide transport, macromolecule metabolic process, lipid transporter activity, glyoxylate cycle, regulation of protein serine/threonine phosphatase activity, and glutathione metabolic process. Figure 6E indicates that the analysis of the downregulated genes revealed nine functional clusters, including RNA metabolic process, photosynthesis, light harvesting in photosystem I, glucan catabolic process, porphyrin-containing compound biosynthetic process, structural constituent of cytoskeleton, transport, macromolecule metabolic process, and protein glutathionylation. According to the results, PVY suppresses the expression of a significant cluster of metabolism-related genes while activating a major cluster of defense function genes in YouJin.

### 3.6 Candidate gene and RT-qPCR verification

**Figure 7:**
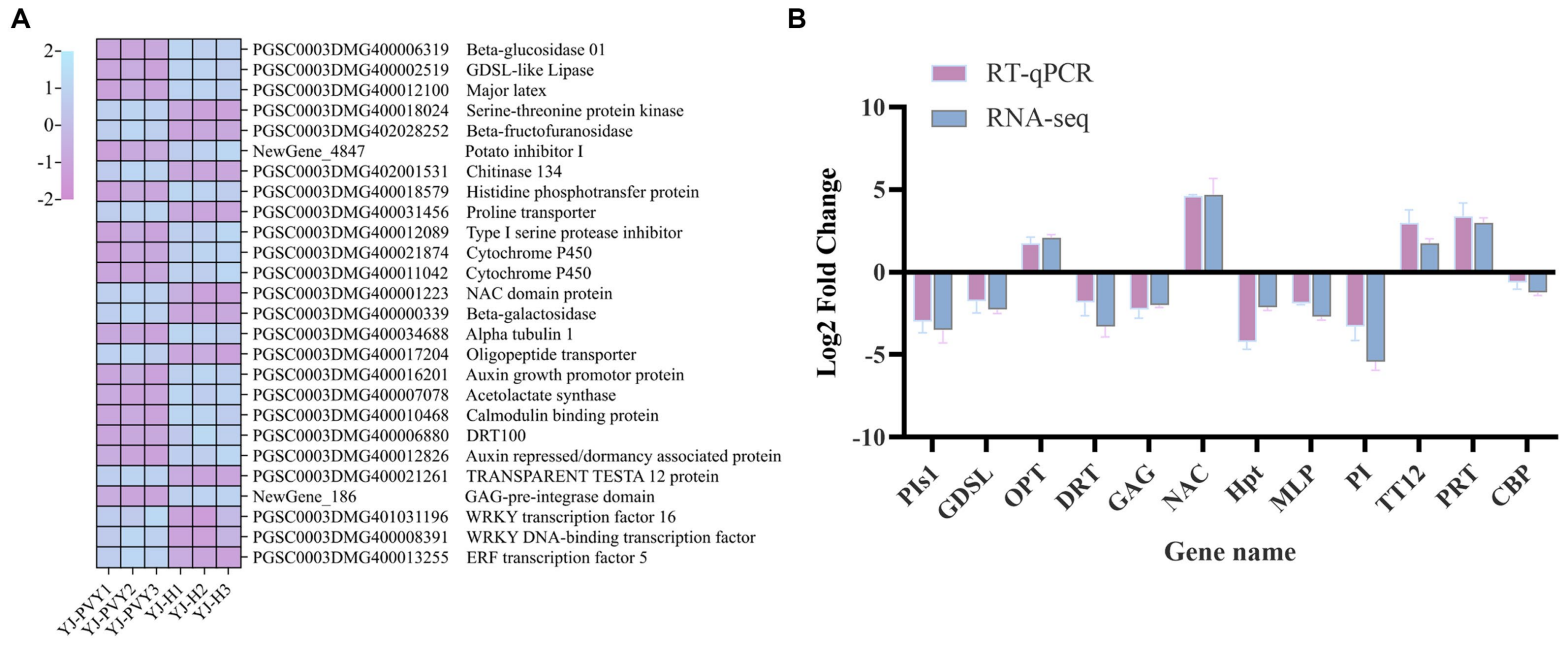
A heat map of the candidate gene and RT-qPCR validation. **(A)** A heat map of the transcript levels of the 26 DEG candidates; relative expression levels are shown as a color gradient from low (purple) to high (blue); **(B)** verification of differentially expressed genes by real-time fluorescence quantitative PCR, including GDSL-like lipase (GDSL), major latex protein (MLP), histidine phosphotransfer protein (Hpt), type I serine protease inhibitor (PIs1), oligopeptide transporter (OPT), NAC domain protein (NAC), DNA damage repair/toleration protein (DRT), protease inhibitor (PI), TRANSPARENT TESTA 12 protein (TT12), proline transporter (PRT), calmodulin-binding protein (CBP), and GAG-pre-integrase domain (GAG).

In this study, we first identified significant DEGs based on their Log_2_FC and −Log_10_FDR values. Then, we annotated these genes with GO, KEGG, and transcription factors to identify key functions and enriched pathways. The candidate genes screened using the method above include STE, MLP, TT12, β-FFase, Chi134, AuP, CYP450, GUS, TUBA1A, Hpt, PIs1, OPT, AHAS, PI, GAG, NAC, DRT, GDSL, PRT, and CBP. Then, six upregulated genes from the WRKY transcription factor family were identified through PPI analysis, with a focus on the plant–pathogen interaction pathway. The candidate genes were selected based on their high expression levels (PGSC0003DMG401031196 and PGSC0003DMG400008391). Finally, PPI network analysis of all differential genes yielded a key modular gene for the upregulated ERF transcription factor (PGSC0003DMG400013255) as a candidate gene. Based on the aforementioned analysis, we selected 26 candidate DEGs (Figure 7A), 12 of which were verified by RT-qPCR. The FC value is calculated using the 2^–ΔΔCT^ method to determine the Log_2_FC value. The results demonstrated that the expression patterns of the selected 12 genes were consistent with the transcriptome data when compared with RNA-Seq (Figure 7). This finding suggests that the transcriptome data are reliable.

## 4 Discussion

PVY is a major threat to cash crops and one of the most economically important plant pathogens, causing severe diseases in cultivated hosts such as potato, tobacco, tomato, and chili, as well as ornamentals (Quenouille et al., 2013). In this investigation, YouJin plants exhibited various symptoms upon PVY infestation, including crumpled plant veins, plant dwarfing, necrotic spots, crumpled and shriveled leaves, deformities, downturned leaf margins, and stunted new leaves, all of which largely impinged on the growth and development of potatoes. Due to the significant economic damage caused by PVY to plants in the Solanaceae family, research on the transcriptional response of these plants to PVY infection remains a research topic of great interest (Guo et al., 2017, 2023; Gutierrez Sanchez et al., 2020; Xu C. et al., 2023; Xu S. et al., 2023). In this study, we analyzed the transcript levels of YouJin in response to PVYros infestation, using RNA-Seq technology to investigate differential gene expression.

The infestation of PVYros changes the YouJin’s performance and regulates related gene expressions. The obtained data identified candidate genes that are likely responsible for the pathogenesis mechanism of PVYros in the potato cultivar YouJin at different levels.

Through the analysis of significant differences in genes, it was found that the GUS gene possessed the highest significance in the signal transduction function of BP (FDR = 2.05E-69) and was downregulated by log2(FC) = −2.07. GUS plays a crucial role in various aspects of plant physiology, such as the synthesis of intermediates in cell wall lignification and the activation of phytohormones and chemical defense compounds. Many plant defense compounds are stored in an inactive glucosylated form. The defense compounds are subsequently activated by GUS when cell fragmentation occurs (Morant et al., 2008).

In the BP response function to stimuli, it was observed that the PIs1 gene was downregulated by log2(FC) = −3.51. Similarly, the PI gene showed downregulation by log2(FC) = −5.42. Protease inhibitors are commonly found in tubers and plant seeds and are believed to act as storage proteins and defense mechanisms. They regulate the activity of proteases, which are crucial for the growth and development of organisms. Protease inhibitors play a significant role in safeguarding plant tissues from pest and pathogen attacks using antinutritional interactions (Kim et al., 2009). Osmani et al. performed a transcriptome screen to identify aspartate protease inhibitor 5 (API5)-resistant cultivars that show significant induction during virus infection. The authors constructed a vector with the potato StAPI35 gene under the control of the cauliflower mosaic virus (CaMV) 5S promoter. Transgenic plants expressing StAPI5 demonstrated significant viral resistance in comparison to the control, indicating that the StAPI5 gene is imperative in enhancing plant resistance to viral stress (Osmani et al., 2022). It is possible that the downregulation of certain plant defense genes during the BP may contribute to PVY infestation.

The elevated differential gene STE [log2(FC) = 3.26] underwent KEGG pathway screening, and, as demonstrated by the MF enrichment diagram, a majority of the protein kinase genes were upregulated. Protein kinases exhibit various functions, including a defensive role such as mitogen-activated protein kinase (MAPK) (Meng and Zhang, 2013), as well as promote viral invasion in plants by phosphorylating activated viral proteins. For instance, PKA phosphorylates BBSV CP in a cAMP-dependent manner to enhance viral particle assembly and stability (Zhao et al., 2015). Similarly, BSMV relies on chl-PGK and Ser/Thr kinase proteins for replication and intercellular movement (Cheng et al., 2013). It was hypothesized that the upregulated Ser/Thr protein kinase genes may improve viral replication during PVY infestation in YouJin.

A NAC transcription factor family of DEGs, which showed a significant upregulation [log2(FC) = 4.69], was compared to a transcription factor database. NAC TFs are known to regulate plant development, senescence, morphological changes, and abiotic stress tolerance (Singh et al., 2021). Studies have also highlighted NAC’s ability to interact with viruses and enhance plant survival under stress when viruses infect host plants. For example, the structural domain transcription factor ATAF2 of *Arabidopsis thaliana* NAC is responsive to TMV infections, and its overexpression leads to a significant reduction in virus accumulation (Wang et al., 2009). The Turnip crinkle virus (TCV) has been demonstrated to interact with a NAC transcription factor, TIP, of *Arabidopsis thaliana*, through its CP. It has been established that TIP expression influences the rate of TCV accumulation in Arabidopsis and that the magnitude of TIP expression influences correct signaling in the SA pathway and other defense responses (Donze et al., 2014). Currently, it is feasible to specifically enhance plant traits by genetically altering NAC transcription factors. In-depth examinations of the functions of NAC transcription factors are hugely important for the advancement of plant molecular breeding (Bian et al., 2020).

We obtained WARK transcription factor family-related genes by PPI analysis of the plant–pathogen interaction pathways. The importance of WRKY TFs in plant defense regulation is well established (Freeborough et al., 2021). WRKYs are involved in both Toll-interleukin-like receptor (TIR) and non-TIR resistance genes, which encode proteins containing NBS-LRR domains (Tarr and Alexander, 2009). Several transcriptome analysis studies have shown that WRKY can also respond to viral infection through conventional signaling pathways, making it a key determinant of viral accumulation. A comparative transcriptome analysis was conducted on TYLCV-resistant and susceptible tomato varieties, Zheza-301 and Jinpeng-1. The analysis revealed that six group III WRKY TFs were upregulated after TYLCV infection. Silencing of WRKY41 or WRKY54 in Zheza-301 resulted in reduced TYLCV DNA accumulation (Huang et al., 2016a). During the South African cassava mosaic virus (SACMV) infection, MeWRKY27 and MeWRKY55 were upregulated at every time point in the susceptible variety T200. Additional Gene Ontology (GO) analysis of Arabidopsis homologs suggests that these MeWRKY TFs may regulate hormone signaling in plants and could play a role in determining plant resistance to biotic stresses (Freeborough et al., 2021).

We conducted a module analysis of DEGs by building a protein–protein interaction (PPI) network. Our KEGG pathway enrichment analysis highlighted the downregulation of pathways for important modular genes, specifically those associated with LHC proteins in photosynthesis (Stare et al., 2015). Photosynthesis is essential for plant growth, development, and potato yield. Cultivars often maintain high growth vigor and tuber yield by upregulating photosynthesis and protein biosynthesis-related proteins (Cheng et al., 2022). The photoprotective functions of LHC proteins include acidification of the lumen of the cystoid body, regulation of chlorophyll and carotenoid synthesis, and the repair of PSI and PSII. LHC proteins act as an adversity condition guardian of photosynthesis (Levin and Schuster, 2023). PVY infestation had a severe impact on YouJin yield due to the inhibition of photosynthesis. The PPI key module network identified AP2/ERF-ERF as a significantly upregulated transcription factor, which has been reported to show an increase in gene expression level after tomato yellow leaf curl virus (TYLCV) infestation. Moreover, the interaction network suggests that AP2/ERF-ERF can interact with MAPK (Huang et al., 2016b). Meanwhile, AP2/ERF-ERF transcription factors regulate multiple plant metabolite biosynthesis (Feng et al., 2020). Additionally, various genes related to ET synthesis and signaling have been reported to be upregulated in potatoes infected with PVY at 6 dpi (Baebler et al., 2014). It is hypothesized that AP2/ERF-ERF may be involved in the regulation of key metabolic pathways in plants after PVY infection to resist virus infection. The results of the functional network analysis showed PVY infection-induced upregulation of plant defense genes, but they also demonstrated that PVY infection also severely disrupted essential metabolic processes and suppressed disease-resistance genes such as glutathionylation and peroxidase activity.

In this study, we only monitored the gene changes in the middle of PVY infection, and there was no temporal gradient, which is a limitation. This period was chosen because it falls within the middle stage of infestation, when PVY symptoms are clearly visible. Additionally, we observed that the majority of genes in the key disease-resistance pathway of YouJin are upregulated during this period, which suggests that this is a critical period for YouJin to resist the infestation of PVY. The DEGs are more representative of this stage. The functional network diagram revealed major genes in function enrichment clusters of upregulated and downregulated genes. Based on this finding, we hypothesized that PVY may further infest by inhibiting key metabolic processes in YouJin. After only 30 days post-inoculation, PVY had significantly affected the photosynthesis of YouJin, which could severely limit its yield. We screened key differential genes from multiple perspectives and obtained the hub genes by constructing a PPI network, which can enrich the candidate genes for potato disease-resistance breeding and improvement and has important research significance.

## Data availability statement

The datasets presented in this study can be found at the NCBI BioProject repository, accession number PRJNA1050800 (https://www.ncbi.nlm.nih.gov/bioproject/?term=PRJNA1050800).

## Author contributions

TY: Data curation, Software, Visualization, Writing – original draft, Investigation, Methodology, Formal analysis. XZ: Conceptualization, Data curation, Investigation, Methodology, Writing – review & editing, Validation, Formal analysis, Resources. JB: Investigation, Methodology, Software, Supervision, Writing – review & editing. WL: Writing – review & editing, Investigation, Methodology. QC: Writing – review & editing, Investigation, Resources. JH: Writing – review & editing, Conceptualization, Investigation, Methodology, Resources. GL: Writing – review & editing, Conceptualization, Investigation, Methodology, Resources. YZ: Writing – review & editing, Conceptualization, Data curation, Investigation. HYZ: Writing – review & editing, Conceptualization, Funding acquisition, Investigation, Project administration, Supervision. MZ: Writing – review & editing, Conceptualization, Data curation, Formal analysis, Funding acquisition, Investigation, Methodology, Project administration, Supervision. HLZ: Writing – review & editing, Conceptualization, Data curation, Formal analysis, Project administration, Supervision, Validation.

## Footnotes

^1^ https://www.chiplot.online/
