# Supplementary material for: Transcriptome analysis of genes involved in the pathogenesis mechanism of potato virus Y in potato cultivar YouJin
Source: Front Microbiol. 2024 Mar 6;15:1353814. doi: 10.3389/fmicb.2024.1353814 (PMC10951100; doi:10.3389/fmicb.2024.1353814)
Supplement: Supplementary file 9 [file Data_Sheet_1.PDF]

Table S1. Primer information of qRT-PCR

Name of primers	Sequence of primer	Size of primers(nt)	Size of expected fragments(bp)
DSL-F	CAAAAAGCGGCATCCATCCT	41	172
GDSL-R	GGCCATACAAATCCTTGACGG		
MLP-F	TGCCCAAATAGAAGTGAAGGCT	42	89
MLP-R	GGACAGGACACATGGTGGAG		
PI-F	TGGAGGGAAAGAGTATGCTCAA	42	189
PI-R	GCATCCCAACAAGTTCAGGC		
Hpt-F	TGGAGGGAAAGAGTATGCTCAA	42	189
Hpt-R	GCATCCCAACAAGTTCAGGC		
PRT-F	GGGCAGAGCCTCAAGTTCATT	41	191
PRT-R	GATCCTGCAGTTGTGCAAGC		
PIs1-F	CTGAATCTTGGTGCAAAGGA	40	140
PIs1-R	CAATGGGACTGGAGAACCAC		
NAC-F	ACCAAGCTGAAGTTTGGACCC	43	118
NAC-R	GCTTTAGAACTTGCATCCCCAA		
CBP-F	CGTATTCCCACCAGCAAACC	40	185
CBP-R	GCTGGCACTTCTAACGGACA		
OPT-F	GCATATATCTATTGGGTATGTCACT	45	156
OPT-R	CGACGGCTAAGGTGTAGAGT		
DRT-F	CTCAACGGGTTGCCGACATT	41	141
DRT-R	CGGCGACGATGAGTGTAGTGA		
TT12-F	AGCTAGAGAAGCCGGTGTAT	41	122
TT12-R	AACAGAAAGCCAGAGGCATGA		
GAG-F	AATGTTGTGGCTGGACCGTT	43	195
GAG-R	ACATCAGAAACAACACTAGCAGA		
